# Comprehensive analysis of five long noncoding RNAs expression as competing endogenous RNAs in regulating hepatoma carcinoma

**DOI:** 10.1002/cam4.2468

**Published:** 2019-08-08

**Authors:** Xin Lou, Jun Li, Dong Yu, Ya‐Qing Wei, Shuang Feng, Jin‐Jin Sun

**Affiliations:** ^1^ Department of Hepatopancreatobiliary Surgery, Tianjin Medical University Second Hospital Tianjin Medical University Tianjin China

**Keywords:** ceRNA, hepatoma carcinoma, lncRNA, prognosis, TCGA

## Abstract

Liver cancer is the most common cancer and is the epitome of a recalcitrant cancer. Increasing evidence shown that long noncoding RNAs (lncRNA) were associated with cancer‐related death and could function as a competing endogenous RNA (ceRNA). To explore regulatory roles and potential prognostic biomarkers of lncRNA for liver cancer, RNA‐sequencing expression data were downloaded from the TCGA database and GEO database. A total of 357 patients were randomly divided into a discovery group and a validation group, of which 313 patients can obtain clinical data. In discovery phrase, 58 lncRNAs, 16 miRNAs, and 34 mRNAs were screened to construct the ceRNA network based on 252 patients employed from discovery group. Univariate and multivariate Cox hazard regression analysis model revealed that five lncRNAs (AATK‐AS1, C10orf91, LINC00162, LINC00200, and LINC00501) from 58 lncRNAs were formulated to predict the overall survival (OS). We used the value of gene expression and regression coefficients to construct a risk score based on the five lncRNAs. Next, we validated our model in the GSE116174 dataset (n = 64) and the validation group (n = 94) from TCGA database. Subgroup analysis suggest that the five lncRNAs played critical parts in early stage in cancer from both discovery and validation groups. The five lncRNAs were also found to be associated with immune cells infiltration including CD4^+^ memory activated, NK cells activated and mast cells activated, then the results were also validated according to the validation group. Furthermore, KEGG pathway enrichment analysis showed that these nine coexpressed modules using the method of WGCNA, and many of these pathways are associated with the development and progression of disease. At last, the transcription factor binding sites (TFBS) of the five lncRNAs were predicted, which help us to understand the potential mechanism that the TFBS adjusted the ceRNA network. In summary, the ceRNA regulatory network may contribute to a better understanding of liver cancer mechanism and provide potential prognostic biomarkers and therapeutic targets.

## Graphical abstract

lncRNA play critical roles in the development of cancer and may have close relation to prognosis. we constructed a ceRNA network, and a risk‐score model based on eight‐lncRNA to predicted the OS time of HCC patients.

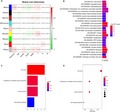

## 1 INTRODUCTION

Hepatocellular carcinoma (HCC) became the sixth common cancer and the death rate increased year by year.^1^ And the rate accounts for a higher proportion in developing countries including China due to the high prevalence of chronic hepatitis C.^2^ There currently exists a lot of ways to treatment including surgical resection, transplantation, and local ablation for early liver cancer,^3^ but the overall survival did not present apparently variation. With the development of molecular multi‐kinase inhibitors, sorafenib, regorafenib, and lenvatinib have increased the overall survival (OS) rate of HCC and have been approved by the US Food and Drug Administration (FDA) to cure HCC.^4^ However, those drugs can only improve less than 4 months OS in advanced HCC and did not response a better prognosis.^4^, ^5^ Therefore, the development of novel treatment, the identification of new prognostic biomarkers and a clearer understanding of molecular mechanisms are essential and urgently required.

Noncoding RNA (ncRNA) sequences include small nucleolar RNAs, long noncoding RNA (lncRNA), miRNA, and small interfering RNA (siRNA), of which lncRNAs are >200 nucleotides in length and regulate gene expression at the levels of chromatin organizational, transcriptional, or posttranscriptional.^6^ miRNAs‐gene‐regulatory ncRNA could direct RNA‐induced silencing complex (RISC) miRNA response elements (MRE), which repressed protein production through inhibiting translation or destabilizing the mRNA.^7^ MRE located in 3′ untranslated region (UTR), coding sequence (CDS), and 5′UTR, and could be found on lncRNA and mRNA. As known, each miRNA has various RNA targets, which has led to the hypothesis that the different RNAs sharing the same MRE compete with each other for limited miRNA,^8^, ^9^ so that acting as competitive endogenous RNAs (ceRNA) and regulating gene expression. LncRNAs are extensively targeted by miRNAs through 3′ UTR or 5′UTR, meaning that they could serve as ceRNAs,^10^ the lncRNA as ceRNA were associated with cellular biological process and also serve important roles in tumorigenesis.^11^ Recent published studies confirmed the ceRNA theory involved in the progression of various types of cancer.^12^, ^13^, ^14^, ^15^ These studies aimed at some genes including mRNA, miRNA, and lncRNA associated with the cancer and confirmed that some of genes serve important roles in treatment and prognosis. With the development of experimental studies and techniques for lncRNA discovery, lncRNA‐associated ceRNA networks have been constructed and analyzed in colorectal cancer, gastric cancer, and osteosarcoma.^13^, ^16^, ^17^ However, there existed fewer analysis of ceRNA network in hepatocellular carcinoma (HCC).

In the present study, we conducted a comprehensive analysis of mRNA, lncRNA, and miRNA expression profiles in HCC and the lncRNA‐sequencing (lncRNA‐seq) data, mRNA‐seq, and miRNA‐seq expression of HCC samples were downloaded from TCGA database and the differently expressed RNAs were screened to construct ceRNA network. Furthermore, univariate and multivariate Cox regression analysis was further conducted to establish a risk assessment system based on the regression coefficient. Subsequently, the assessment model was validated in validation and entire group, and we further explore biological function as well as immune cells infiltration characters associated with the five lncRNAs, then we predicted the TFBS of HCC which may regulate the ceRNA network to understand the potential mechanism.

## 2 MATERIAL AND METHOD

### 2.1 Patient information and preprocessing

The data of RNA‐seq expression and clinical information were downloaded from the TCGA database (https://portal.gdc.cancer.gov/) and GEO database (GSE116174). The patients obtained from TCGA were randomly divided into a discovery group and a validation group. The data downloaded from GEO were used as a validation group. The discovery group was used to construct model, and the validation group was used to validate the efficiency of the model. Firstly, we obtained the lncRNA expression based on annotation of Genecode (https://www.gencodegenes.org/) by screening them from the mRNA expression profiles we have downloaded, Consequently, the RNA‐sequencing data of TCGA covered 19767 mRNA, 14718 lncRNA, and 1881 miRNA. Next, after we conducted normalization of RNA‐seq of TCGA data and GEO data, the differentially expressed mRNAs (DEmiRNA), lncRNAs (DElncRNA), and miRNA (DEmiRNA) were conducted based on Bioconductor package of edgeR in R 3.5.2 with the threshold of |log2 fold change|>2 and *P* < .01.

### 2.2 Construction of ceRNA network

The miRcode was used to predict the interaction of DElncRNA with DEmiRNA, and the mRNAs were retrieved according to miRTarBase, TargetScan, and miRDB based on targeted miRNA. To increase the reliability of the results, only miRNA‐mRNA interaction found in all three databases were selected as candidate genes for constructing the ceRNA network. Then, the obtained mRNA intersected with the DEmRNAs to screen final targeted mRNAs. Next, the lncRNA‐miRNA‐mRNA ceRNA network was constructed. At last, the interactions and visualization were conducted by the Cytoscape software (https://cytoscape.org/).

### 2.3 Risk assessment model construction and evaluation

After ceRNA network was constructed, we obtained lncRNA to univariable Cox regression analysis to select lncRNA associated with OS of patients with liver cancer, only the lncRNA with a statistical significance (*P* value <.05) were enrolled into multivariable Cox regression. A patient risk assessment model was constructed through the regression coefficients with lncRNA expression. In other words, the risk score was the linear combination of expression value of selected lncRNAs weighted by the regression coefficients. A risk score of patients was calculated based on the Equation. Risk score = Exp1 * Coe1 + Exp2 * Coe2 + Exp3 + Coe3 + ……Expi * Coei.^18^ In this Equation, the Exp are the expression value of lncRNAs and Coe are their corresponding coefficients from the multivariable Cox regression analysis.

### 2.4 Prognostic survival analysis

The risk scores of HCC patients were calculated according to above risk assessment system, the patients were divided into high risk and low risk using the median risk score as boundary. The Kaplan‐Meier method was used to assess the efficiency on OS in high‐risk and low‐risk patients. The *P* value of log‐rank test less than .05 was considered as significance. We conducted the prognostic survival analysis on all this three groups including discovery group, validation group from TCGA, validation group from GEO. Then, we performed an entire analysis to combine the all sample from these three groups.

### 2.5 Pathway enrichment analysis according to weighted correlation network analysis (WGCNA)

We conducted a coexpression network using Bioconductor package of “WGCNA” in R 3.5.2 to find the gene modules closed to our risk scores. The thresholding power was selected to 5 and the genes were clustered into nine modules based on clinical characters. The most significant modules associated with risk score were selected and the genes enrolled into this module were used to conduct biology processes analysis and pathway enrichment analysis. The biology processes analysis was performed using the online web tool “DAVID” (https://david.ncifcrf.gov/). The pathway enrichment analysis was performed according to the Bioconductor package of “clusterProfiler” in R 3.5.2.

### 2.6 Evaluation of tumor infiltrating immune cells and the relation of immune cells with five lncRNAs

To infer the infiltrating immune cells associated with five lncRNAs, we used the targeted mRNAs to predict the proportion of 22 types of infiltrating immune cells using the CIBERSORT web portal (https://cibersort.stanford.edu/index.php) which is a gene expression‐based deconvolution algorithm.^19^ We can obtain the significant immune cell type and the difference in immune cells between cancer and normal tissue, and we further study the relation of these immune cells with our model to evaluate the OS in patients with HCC.

### 2.7 Transcription regulation prediction on ceRNA network

To further understand the mechanisms of ceRNA network in HCC, we predict transcription factor binding sites (TFBS) which regulate the ceRNA network in HCC. We search the promoter region of gene on the basis of the web tool “NCBI.” At first, the potential promoter region is generally thought the region from the sequence of 2000 bp upstream to 100 bp downstream of the starting gene point. Then, the TFBS would be predicted using web tool “UCSC” and “JASPAR.” The intersections of TFBS among the ceRNA network including lncRNA associated with prognosis were calculated respectively, the TFBS were thought to regulate ceRNA network. At last, we conducted KEGG pathways analysis based on these TFBS, which help us to better understand the mechanism of the ceRNA with TFBS.

## 3 RESULTS

### 3.1 Data source and identification of DERNAs

**Figure 1:**
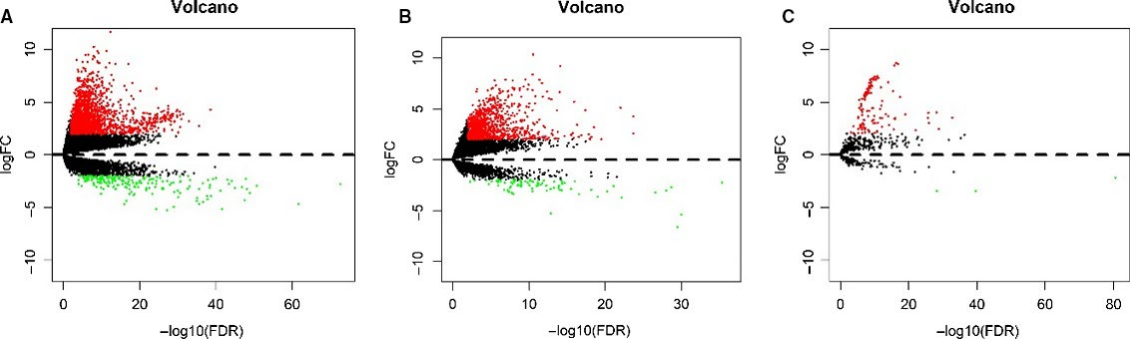
A Volcano plot of differentially expressed RNAs in patients with hepatocellular carcinoma(|log2FC| > 2 and *P* < .01). A, DEmRNAs; B, DElncRNAs; C, DEmiRNA. up‐regulated RNA and down‐regulated was represented in red dot and green dot respectively

A total of 357 patients with liver cancer and 14748 lncRNA expression values were collected from TCGA database. Patients were randomly divided into a discovery group (n = 252) and validation group (n = 105). The discovery group including 33 normal and 219 tumor tissue patients obtained 1035 DElncRNAs according to the criteria (*P* < .01 and |log2FC| > 2). The volcano plot is presented in Figure 1. A total of 123 DEmiRNAs and 1986 DEmRNAs were obtained from TCGA database. And the results are shown in Figure 1.

### 3.2 Construction of lncRNA‐miRNA‐mRNA ceRNA network

**Figure 2:**
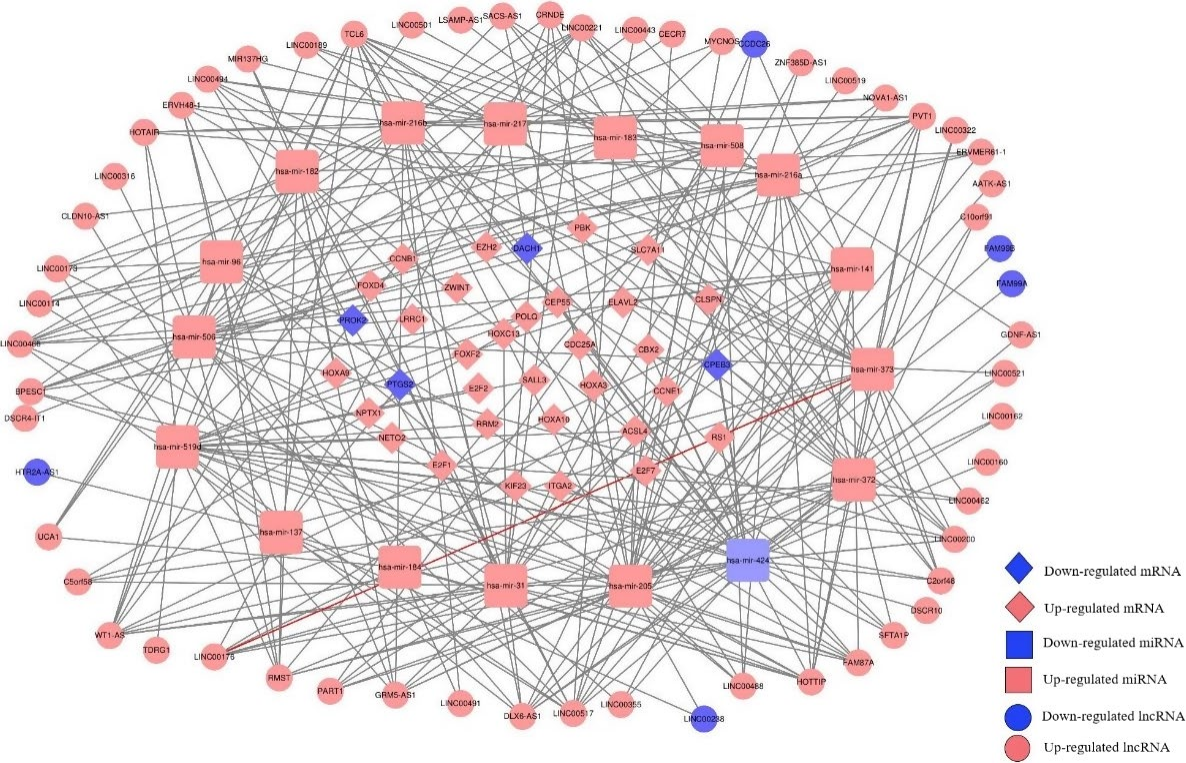
The visualization of ceRNA network in hepatoma carcinoma. The lncRNA‐miRNA‐mRNA ceRNA network

We assessed the relationship between miRNAs and DElncRNAs on basis of miRcode downloaded from the website (http://www.mircode.org/) which present correspondence between lncRNAs and miRNAs. The target mRNA of DEmiRNA was predicted according to the intersection of these three databases (TargetScan, miRDB, and miRarBase). At last, 58 lncRNAs, 16 miRNAs, and 34 mRNAs were included to construct ceRNA network, and the visualization of coexpression was built using the software of Cytoscape (Figure 2).

### 3.3 Screening for lncRNAs as biomarkers related to overall survival and prognosis

**Figure 3:**
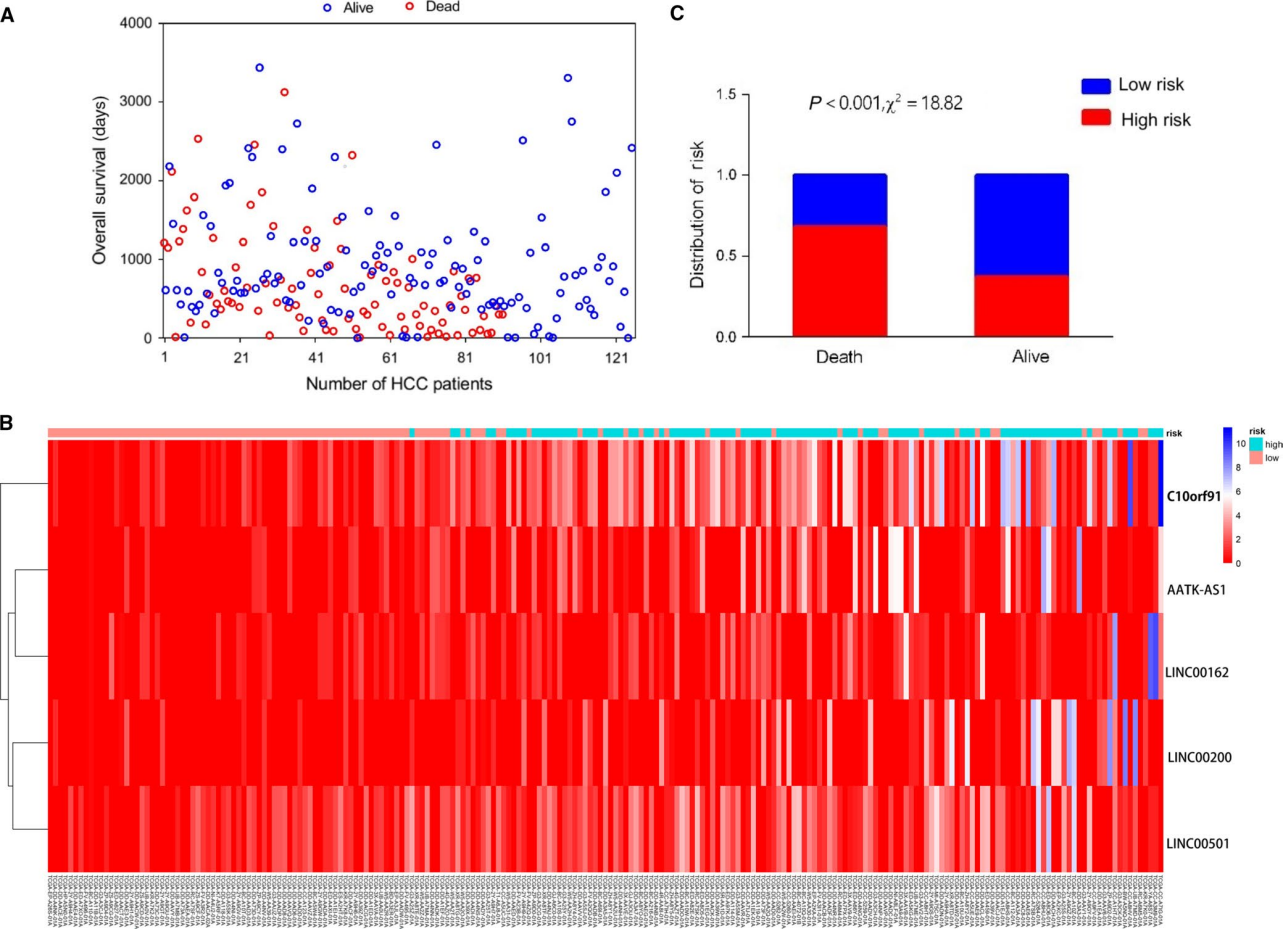
The five‐lncRNA model and its prognostic value for liver cancer. A, survival time of patients with different vital status. B, Heatmap of five lncRNAs expression between low‐risk score and high‐risk score. C, The risk attribution in Death group and Alive group

Based on the ceRNA network, a total of 58 differentially expressed lncRNA were analyzed by the Univariate and Cox hazards regression analyses. Twenty‐four lncRNAs were identified to be significantly corrected with prognosis based on univariate hazards regression analysis (*P* value <.05), of which these lncRNAs were screened to conduct multivariate Cox regression analysis. We finally obtained five lncRNAs, namely, AATK‐AS1, C10orf91, LINC00162, LINC00200, and LINC00501 (Table 1). On the basis of multivariable Cox, a risk score was constructed as following: risk score = 1.178 × exp(AATK‐AS1) + 1.181 × exp(C10orf91) + 1.278 × exp(LINC00162) + 1.271 × exp(LINC00200) + 1.198 × exp(LINC00501). The heatmap revealed that the expression level of five lncRNAs varied as the risk scores (Figure 3). Our data showed that mortality rate in high‐risk group was significantly higher than low‐risk group (Figure 3), which indicates the five lncRNAs play a critical role in liver cancer.

**Table 1:** Five lncRNAs significantly associated with the overall survival in patients with liver cancer in the discovery group

Gene name	Ensembl ID	Univariate analysis		Multivariate analysis	
		HR	*P* values	HR	*P* values
AATK‐AS1	ENSG00000225180	1.153	.021	1.178	.016
C10orf91	ENSG00000180066	1.247	<.001	1.181	<.001
LINC00162	ENSG00000275874	1.266	<.001	1.278	<.001
LINC00200	ENSG00000229205	1.246	<.001	1.271	<.001
LINC00501	ENSG00000203645	1.191	.027	1.198	.021

### 3.4 The prognostic values of five lncRNAs in discovery and validation group

**Figure 4:**
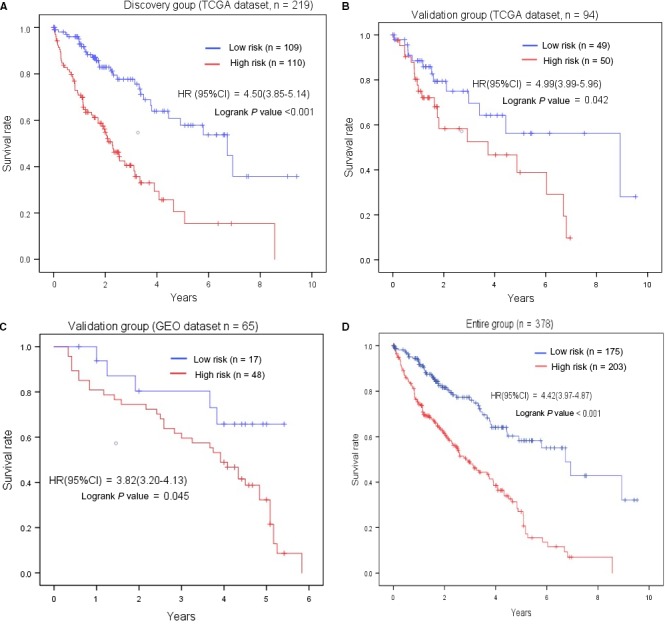
The association between five‐lncRNA signature and overall survival in discovery and two validation groups. Kaplan‐meier survival curves were plotted to estimate the overall survival probilities for the low‐ and high‐risk group in the discovery group (A). B, validation group from TCGA. C, validation group from GEO. D, entire group

Our data in discovery group showed that the patients who had low‐risk scores present a longer OS time than higher risk group (Figure 4A). To validate above finding, we employed the validation group from TCGA and a GSE116174 dataset. The two validation groups were consistent with the result of discovery group. The entire samples were employed together and conducted a survival analysis (Figure 4D), which also confirmed the low‐risk group has a better overall survival compared with high‐risk group. In short, this finding further presents that the five lncRNAs are critical biomarkers which could affect the prognosis of patients with HCC.

### 3.5 The five lncRNAs were associated with OS in patients with cancer at early stage

**Figure 5:**
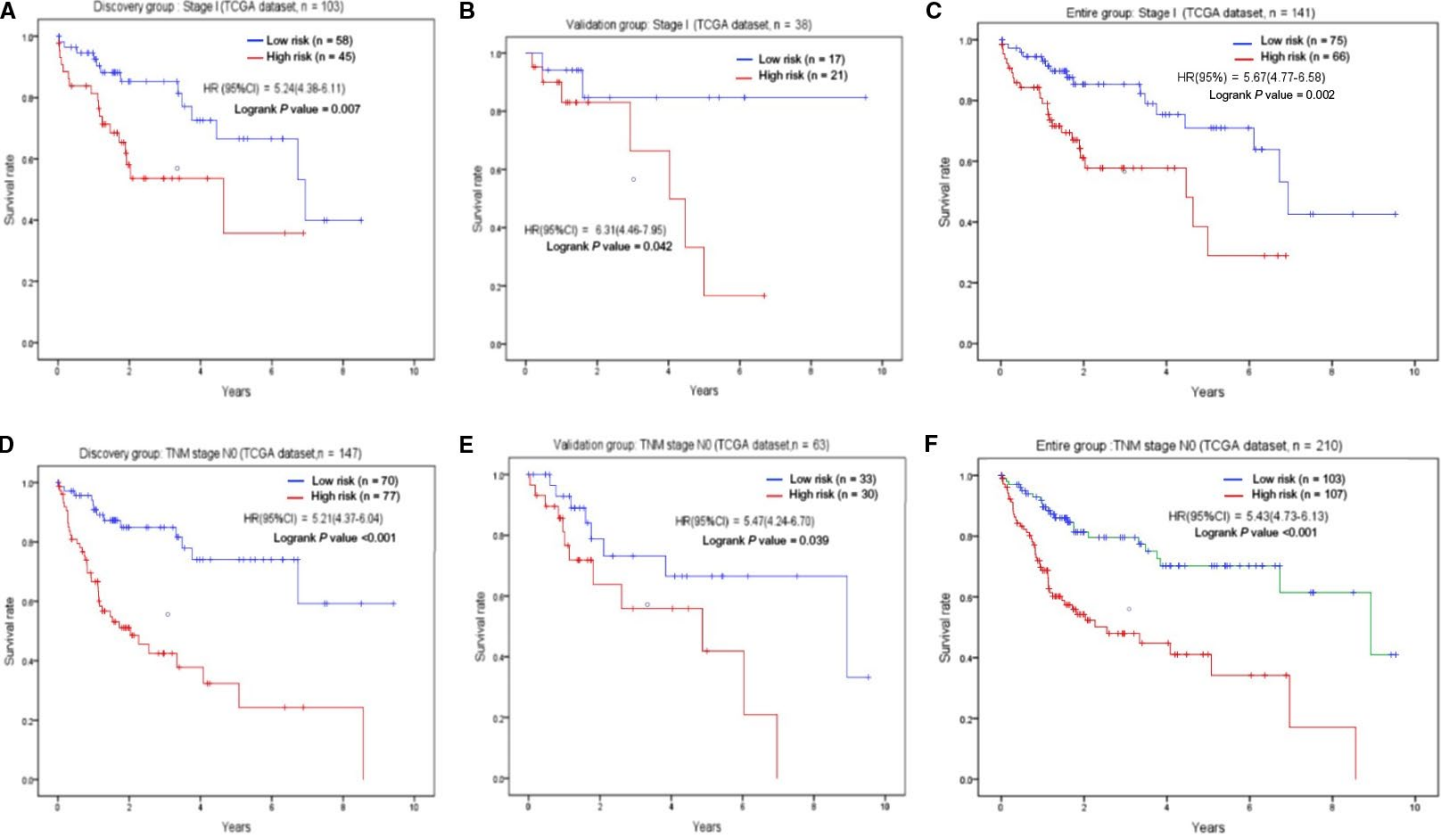
The association between five‐lncRNA and overall survival in patients with early cancer. The survival curves of discovery group with stage I (A), validation group (B), entire group (C) were plotted. The similar results were obtained from the patients with N0 in discovery group (D), validation group (E), entire group (F)

To explore the effects of five lncRNAs on clinical characteristics, we group patients based on five characteristics including age, gender, grade, AJCC stage, and TNM staging. According to the results of analysis, we found the patients with cancer at early stage seem more related with the five lncRNAs (Table 2). As we observed from analysis, the stage I and N0 staging present statistical significance in discover group (*P* < .05). Moreover, we also explore the HR in different stage and found the trend is not statistical significance (*P* for trend >.05), which imply the risk model associated with five lncRNAs may only be related to OS in patients with early cancer. To validate our founding, we further calculated the prognostic values of the five lncRNAs in validation group and entire group (stage I, N0 staging). Kaplan‐Meier analysis method was conducted to visualize the OS between high‐risk and low‐risk groups in patients with early cancer (Figure 5).

**Table 2:** The association between five lncRNAs and overall survival of patients in discovery and validating groups

	Discovery group			Validation group			Entire group		
	Number (high/low)	HR (CI 95%)	*P* values	Number (high/low)	HR (CI 95%)	*P* values	Number (high/low)	HR (CI 95%)	*P* values
Age									
<=60	49/45	5.08 (4.11‐6.06)	.003	19/24	4.12 (3.07‐5.17)	.268	68/69	4.83 (4.05‐5.61)	.006
>60	47/56	4.06 (3.21‐4.91)	<.001	20/25	6.12 (4.73‐7.50)	.707	67/61	4.46 (3.86‐5.38)	<.001
Unknown	14/7	2.44 (1.83‐3.05)	.99	1/5			15/12	3.05 (2.22‐3.89)	.897
Gender									
Male	60/74	4.67 (3.90‐5.44)	<.001	32/30	4.66 (3.80‐5.52)	.898	92/104	4.69 (4.03‐5.35)	.001
Female	50/34	4.02 (3.15‐4.89)	.001	8/24	5.37 (3.74‐7.00)	.766	58/58	4.47 (3.65‐5.28)	.001
Grade									
G1	7/23	4.88 (3.71‐6.05)	.001	6/8	4.17 (2.76‐5.59)	.745	13/31	4.17 (3.72‐5.70)	.014
G2	47/50	5.01 (4.02‐6.01)	<.001	15/23	5.27 (3.86‐6.69)	.460	70/66	5.10 (4.29‐5.92)	.003
G3	36/24	3.98 (2.81‐5.14)	.002	16/16	5.91 (4.14‐7.69)	.187	52/40	4.95 (3.88‐6.02)	.001
G4	20/11	2.78 (2.06‐3.50)	.707	3/7			23/18	2.98 (2.32‐3.65)	.714
AJCC stage									
I	45/58	5.24 (4.38‐5.11)	.007	21/17	6.31 (4.46‐7.95)	.042	66/75	2.34 (1.34‐4.09)	.003
II	20/26	4.42 (2.98‐5.87)	.001	9/18	2.80 (0.50‐6.44)	.362	34/39	2.15 (1.09‐4.25)	.027
III	30/17	3.59 (2.35‐4.82)	.083	9/10	1.84 (0.28‐2.50)	.764	22/44	2.09 (1.02‐4.29)	.043
IV	15/7	2.51 (1.73‐3.29)	.258	5/4	3.07 (0.09‐12.60)	.955	12/19	2.02 (0.69‐5.88)	.195
*P* for trend			>.05			>.05			>.05
TNM									
T									
T1	47/62	5.16 (4.33‐6.00)	.005	21/19	3.25 (2.53‐6.81)	.035	68/81	5.80 (4.90‐6.71)	.006
T2	25/26	4.05 (2.78‐5.32)	.001	11/20	5.86 (4.10‐7.61)	.322	37/45	4.87 (3.80‐5.93)	.039
T3	31/15	3.71 (2.47‐4.94)	.101	9/11	3.26 (2.51‐3.78)	.041	40/26	3.39 (2.53‐4.26)	.151
T4	7/5	1.41 (0.86‐1.96)	.301	5/6	2.98 (2.01‐3.94)	.814	12/11	1.49 (1.01‐1.96)	.518
*P* for trend			>.05			>.05			>.05
N									
N0	77/70	5.21 (4.37‐6.04)	<.001	30/33	5.47 (4.24‐6.70)	.039	107/103	5.43 (4.73‐6.13)	<.001
N1	33/38	3.13 (2.51‐3.75)	.042	14/18	4.53 (3.27‐5.78)	.450	53/48	3.41 (2.82‐3.99)	.241
M									
M0	77/81	4.99 (4.18‐5.80)	<.001	26/39	5.58 (4.42‐6.75)	.119	103/120	5.22 (4.54‐5.89)	<.001
M1	33/27	3.22 (2.51‐3.93)	<.001	14/15	3.33 (1.98‐4.69)	.176	47/42	3.29 (2.66‐3.92)	.028

### 3.6 Some pathways were associated with the five lncRNAs based on method of WGCNA

**Figure 6:**
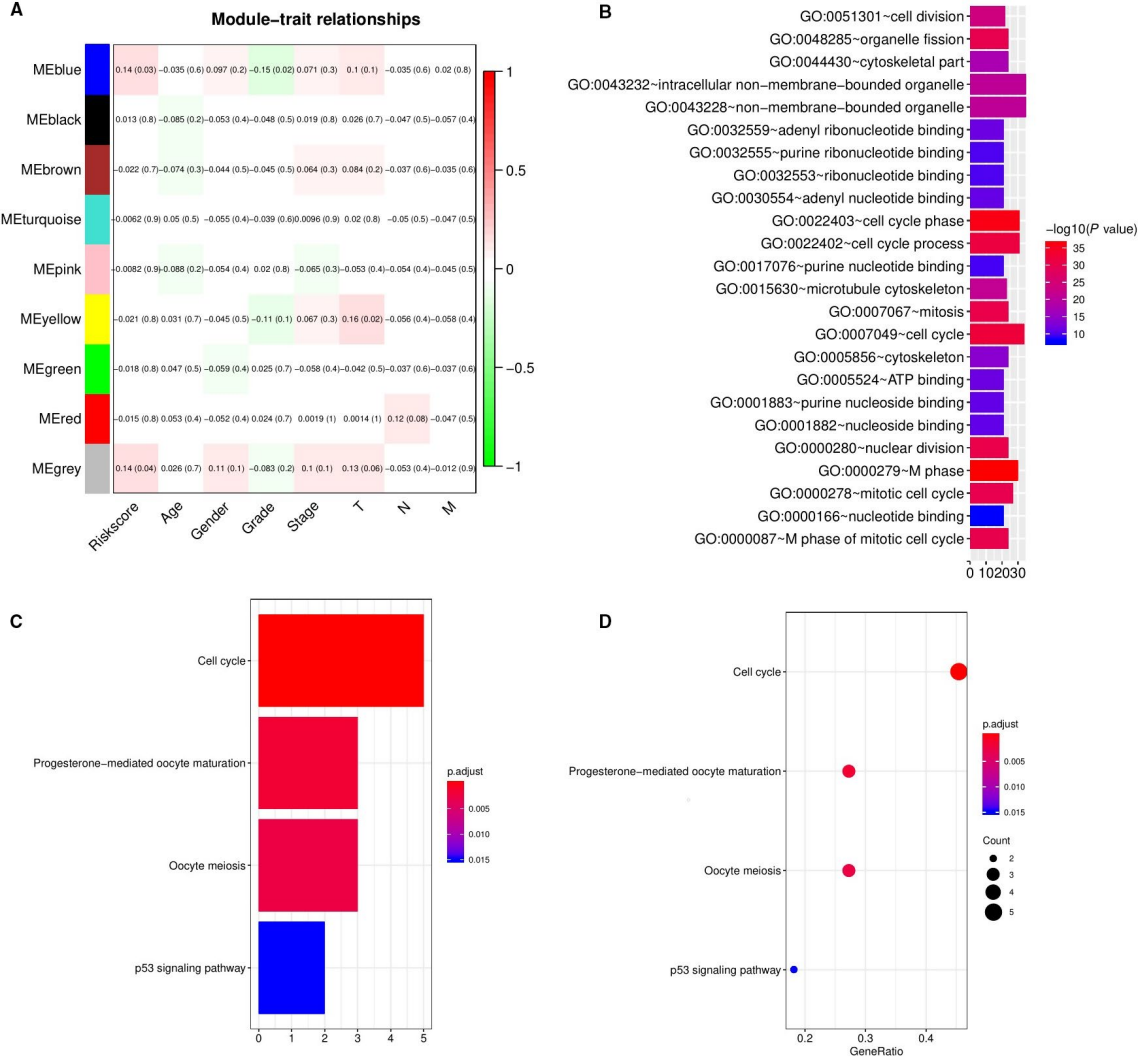
WGCNA predicted GO and KEGG pathways associated with the five‐lncRNA signature. A, the gene clusters obtained by WGCNA method. B, the GO analysis of the co‐expressed genes in blue module. C and D, significantly enriched pathways of the genes in blue module

To further investigate the potential biological functions of the five lncRNAs, we used the WGCNA method to cluster genes based on the data of DEmRNA obtained from TCGA. We identified a total of nine coexpression modules with the threshold of five and found that blue module was positively correlated with the risk score (*P* value = .03), and GO functional biology processes and KEGG pathways enriched analysis using the genes enrolled into blue module are shown in Figure 6. GO functional biology processes include cell division, organelle fission, cytoskeletal part, and so on. KEGG pathway enrichment analysis was then performed on the basis of the package (clusterProfiler) of R. Four pathways enriched by DEmRNAs from ceRNA network include Cell cycle, p53 signaling pathway, Oocyte meiosis, and progesterone‐mediated oocyte maturation, which were related to prognosis of HCC.

### 3.7 The five lncRNAs were associated with immune cells infiltration

**Figure 7:**
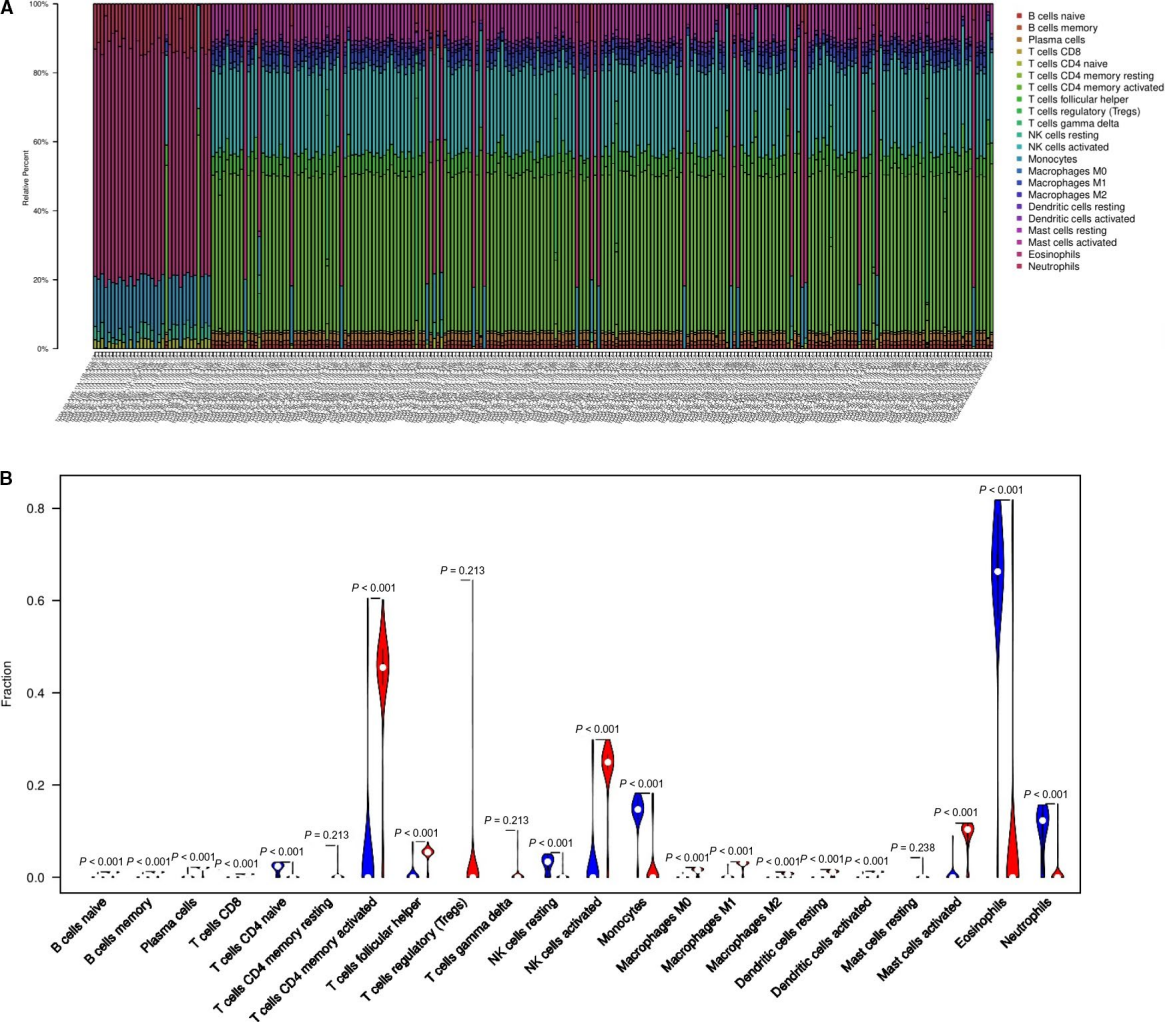
Immune cell composition in liver cancer and healthy livers. A, composition of infiltrating immune cells in different patients. B, vioplot visualizing the differentially infiltrated immune cells in cancer and normal groups on the basis of the 34DEmRNA

**Figure 8:**
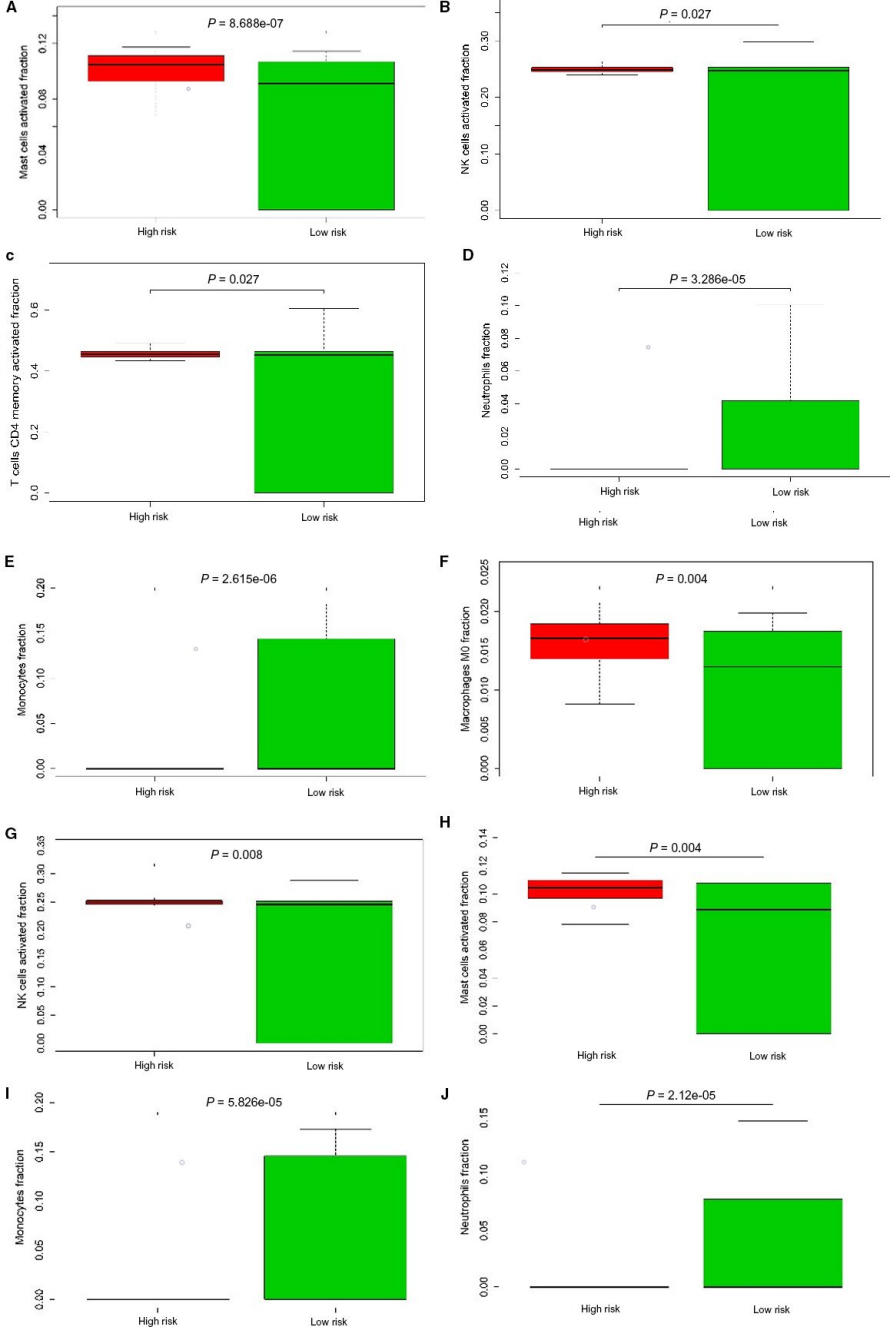
The relation of the immune cells to the five‐lncRNAs. A‐C, CD4^+^ memory activated, NK cells activated and mast cells activated accounted for a higher proportion in high risk group. D and E, Monocytes and neutrophils decreased. In validation group, we obtain the same results (F‐J)

To further explore DElncRNA, we developed CIBERSORT method to search the most significant tumor‐infiltrating immune cells and its correlation with immune cell type in liver cancer related to the DEmRNA (34 mRNA). Based on the CIBERSORT, we found the five lncRNAs played critical in the enumeration and activation status of five immune cell subtypes between paired cancer and normal tissue. Figure 7A summarizes the results obtained from 253 patients. There existed significant variation between normal and tumor group, which indicated the different subpopulations were closely correlation. Compared to normal tissue, HCC tissue related to five lncRNAs contained a higher proportion for T CD4^+ ^memory activated, NK cells activated and mast cells activated (*P* < .05), while the monocytes and neutrophils decreased (Figure 7C). These results indicated that this five lncRNAs played critical role in immune infiltration and the characters with a tightly regulated process may have important clinical meanings. We explored the relationship of risk score with the significant immune cell based on five lncRNAs, the results also confirmed the five lncRNAs has close relation to the five types of immune infiltration cell (Figure 8). Besides, to estimate the accuracy, we also use the validation group to invalidate the results (Figure 8F‐J).

### 3.8 Transcription regulation prediction of ceRNA in HCC

**Figure 9:**
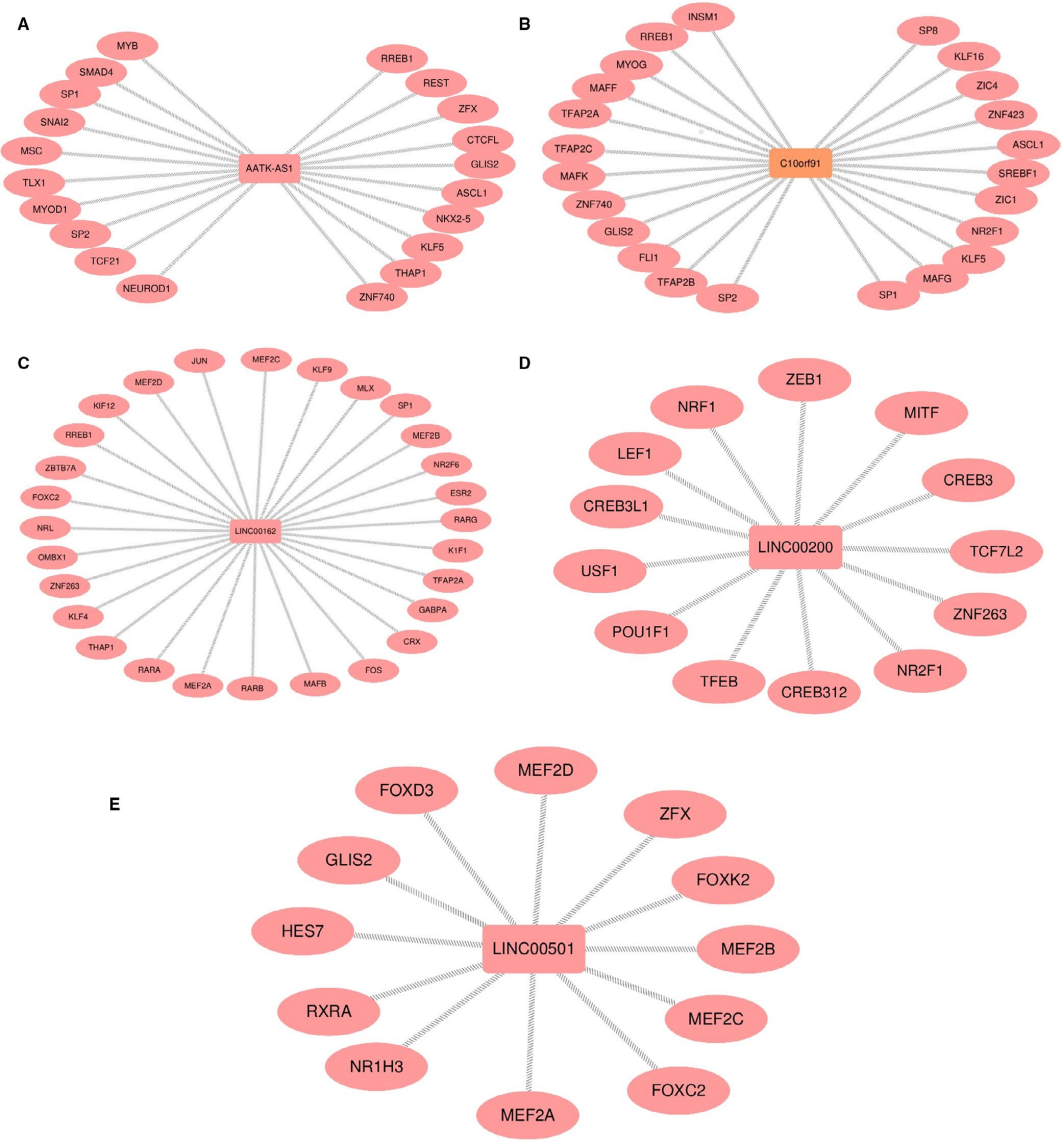
The potential TFBS of five lncRNA were predicted based on the same transcription direction

**Figure 10:**
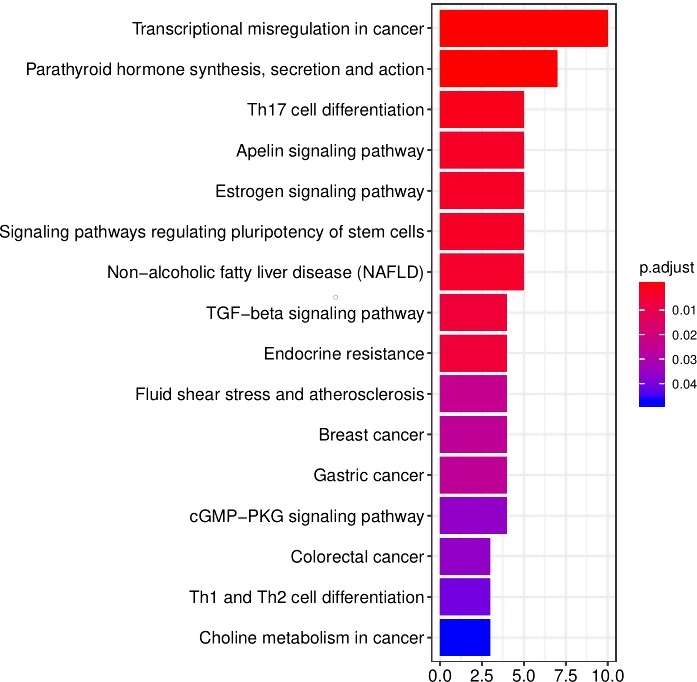
We conducted a KEGG analysis based on the TFBS associated with the five lncRNA

We first got the promoter region of the five lncRNAs, and found most of lncRNAs was located in sense strand except one lncRNA LINC00162 which present reverse transcription. The promoter region was calculated based on transcription direction, respectively. Then, the TFBS were predicted in the promoter region using the web tool “UCSC” and “JASPAR.” Figure 9 present the TFBS of five lncRNAs. As known, the TFBS which present the same transcription direction with target gene has the higher value to explore. We further explore the more significant TFBS, we got a total 96 kinds of TFBS, of which 20 kinds of TFBS were achieved in AATK‐AS1 promoter region, 23 kinds of TFBS in C10orf91 promoter region, 28 kinds of TFBS in LINC00162 promoter region, 13 kinds of TFBS in LINC00200 promoter region, and 12 kinds of TFBS in LINC00501 promoter region (Figure 9). To better understand the mechanisms of TFBS, we conducted a KEGG pathways enriched analysis based on these TFBS associated with the five lncRNAs, and found these TFBS play critical role in transcriptional misregulation in cancer, Endocrine resistance, cGMP–PKG signaling pathway, and so on (Figure 10). These findings predicted the potential TFBS and attributed to understand the mechanisms of the ceRNA network with TFBS in HCC.

## 4 DISCUSSION

The novel hypothesis has been confirmed that each RNA sharing the same MREs could interact with or compete each other, which present a new mechanism of gene expression regulation that could be used to further understand of various diseases including cancer. In the present study, we identified DEmiRNAs, DElncRNAs, and DEmRNA between HCC tissues and normal tissues, we then construct a lncRNA‐miRNA‐mRNA ceRNA network the DElncRNAs in the ceRNA were analyzed for their association with the survival and clinical features of HCC patients, and we conducted validated the association with OS in two independent datasets. Besides, we found the expression of these five lncRNAs presented a statistical significance in patients with early stage.

In our study, we obtained a total five lncRNAs associated with clinical characters in HCC, including AATK‐AS1, C10orf91, LINC00162, LINC00200, and LINC00501. Some of these lncRNAs were reported in cancer for first time including AATK‐AS1 and LINC00200, while C10orf91 LINC00162, LINC00501, and HTR2A‐AS1 has been reported in other type of cancer. Recent studies reported that C10orf91 were related with OS on the basis of complex integrated analysis of lncRNAs‐miRNAs‐mRNAs regulatory network in oral squamous cell carcinoma,^20^ LINC00162 named p38 inhibited cutaneous squamous cell carcinoma associated with lncRNA which promotes growth of cutaneous squamous cell carcinoma by regulating ERK1/2 activity,^21^ the rest of lncRNAs were all related to cell proliferation and contribute to carcinogenesis.^22^, ^23^, ^24^

One of the important findings in this study was that the five lncRNAs were associated with OS in early cancer stages. Our data showed their association reached a high significance in discovery group and in validation group, which imply that the five lncRNAs were a significant prognostic symbol for HCC with early stage. In order to investigate the potential mechanisms on the five lncRNAs, we further conducted bioinformatic analysis. We clustered nine gene modules from 1987 differentially expressed genes based on the method of WGCNA, and found the blue modules was associated with the five lncRNAs. Pathway enrichment analysis further suggested that genes enrolled into blue module were mostly enriched cell cycle and p53 signaling pathway which contributes to enhanced proliferation of breast cancer cells,^25^ indicating that the five lncRNAs could affect cell and consequently contributed to tumor progression. In other word, we may conclude that the five lncRNAs regulated the growth of HCC by regulating p53 signaling pathway. Of course, there still need more further studies to confirm. In short, the five lncRNAs play a major role in influencing prognosis.

The tumor‐related microenvironment including immune cells, fibroblasts, and endothelial cells could make inhibitory effect on malignant cell, but with progression, tumor cells could grow, invasion, even metastasis by circumvent inhibitory signals and immune cells.^26^ There is a complex relation of immune cells and malignant cells in cancer which has high relevance to immune system in either tumor‐promoting or tumor‐inhibiting roles, in present study, we infer the proportions of 22 immune cell from the DEmRNA used to construct ceRNA network using a silicon analysis, known as CIBERSORT.^27^ Then, we conducted comprehensive analysis of clinical impact of immune response in HCC. We further compared the immune cells in high‐risk and low‐risk group. CIBERSORT was always used based on thousands of genes screened from raw data, which means that the hub gene is not clear.^19^, ^28^ Aimed at DEmRNA enrolled into ceRNA network, our data reveal that CD4 memory activated, NK cells activated and mast cells resting compared with normal increased and present statistically significance, while monocytes and neutrophils decreased. Thus, it can be seen that CD4 memory activated cells play a role in the development of HCC, which confirmed that the five lncRNAs have closely relation to immune cells and be a potential therapeutic target. Based on the above immune cells associated with the DEmRNAs, the high‐risk present significant difference compared with low‐risk group, which could validate the relation of five lncRNAs with immune cells.

We could better understand the potential mechanism of the five lncRNAs through the way of predicting the TFBS. The promoter region of the five lncRNAs was predicted using the web tool “NCBI,” then, we can obtain the potential TFBS of the five lncRNA based on the promoter region. The TFBS was screened to further explore which has the same transcription direction to the lncRNA. KEGG enrich pathways were conducted on the screened TFBS to explore further functions. The top pathway was transcriptional misregulation in cancer, and the result was also reported in Lee's study that misregulation of gene expression can cause a broad range of diseases including cancer and participated in cancer progression by regulating cell cycle and cell proliferation.^29^ TGF‐beta signaling pathway was an important role in cervical cancer.^30^ Another study reported suppressing the TGF‐beta signaling pathway was a well‐known immunosuppressor and proangiogenic factor, contributing to fight against cancer.^31^ These studies have reported these function or pathway which might explain the potential mechanism of HCC and provide the further thought to conduct. We assumed that the TFBS might play important roles in regulating the ceRNA in liver cancer.

To conclude, lncRNA play critical roles in the development of cancer and may have close relation to prognosis. We constructed a ceRNA network, and a risk‐score model based on five lncRNAs to predict the OS of HCC patients, which could help people to assess the prognosis of HCC with higher accuracy. The risk association of five lncRNAs was more significant in patients with early stage. We explored the immune cell associated with the five lncRNAs, which contributed to immune therapy. Prediction of TFBS associated with five lncRNAs help us to understand the potential mechanism. It still needs further studies to validate our founding.

## CONFLICT OF INTERESTS

The authors declare no competing interests.

## AUTHOR CONTRIBUTIONS

Xin Lou was involved in writing of this article, data processing, and preparing of figures. Jun Li was involved in data processing. Dong Yu was involved in preparing of figures. Ya‐Qing Wei and Shuang Feng were involved in writing of this article. Jin‐Jin Sun was involved in putting forward the idea. All authors have read and approved the final manuscript.
